# Dosimetric Comparison of Automated Noncoplanar VMAT (HyperArc) Versus CyberKnife for Single-Fraction Vestibular Schwannoma Stereotactic Radiosurgery

**DOI:** 10.3390/cancers18081207

**Published:** 2026-04-10

**Authors:** Zhenyu Xiong, Yin Zhang, Lili Zhou, Keying Xu, Xinxin Zhang, Loren Bell, Fredrick Warburton, David Huang, Sabin B. Motwani, Charles S. Cathcart, Ke Nie, Ning Yue, Xiao Wang

**Affiliations:** 1 Department of Radiation Oncology, Rutgers Cancer Institute, Rutgers, The State University of New Jersey, New Brunswick, NJ 08901, USA; kx57@cinj.rutgers.edu (K.X.); xz562@cinj.rutgers.edu (X.Z.); kn231@cinj.rutgers.edu (K.N.); yuenj@cinj.rutgers.edu (N.Y.); xw240@cinj.rutgers.edu (X.W.); 2 Robert Wood Johnson Medical School, Rutgers, The State University of New Jersey, New Brunswick, NJ 08901, USA; motwansa@cinj.rutgers.edu; 3 Department of Radiation Oncology, RWJBarnabas Health, West Orange, NJ 07052, USA; lili.zhou@rwjbh.org (L.Z.); loren.bell@rwjbh.org (L.B.); fredrick.warburton@rwjbh.org (F.W.); david.huang@rwjbh.org (D.H.); charles.cathcart@rwjbh.org (C.S.C.)

**Keywords:** vestibular schwannoma, stereotactic radiosurgery, HyperArc, noncoplanar VMAT, CyberKnife, dosimetric comparison, high definition MLC

## Abstract

Background: Vestibular schwannoma (VS) stereotactic radiosurgery (SRS) requires high target conformality and rapid dose falloff to spare adjacent organs at risk (OARs), particularly the brainstem. HyperArc (HA) is an automated noncoplanar volumetric-modulated arc therapy (VMAT) approach designed to standardize and streamline cranial SRS planning and delivery. We compared CyberKnife (CK) with HA for single-fraction VS SRS and evaluated the impact of multileaf collimator (MLC) leaf width. Methods: Fifteen VS cases previously treated with single-fraction CK SRS (12.5 Gy) were retrospectively replanned using HA. HA plans used four preconfigured noncoplanar partial arcs and were created with either a standard 5.0 mm MLC (HA-SMLC) or a 2.5 mm high-definition MLC (HA-HDMLC). HA plans were normalized to match the prescription dose target coverage of the corresponding CK plan for each of the patients. Endpoints included planning target volume (PTV) dosimetric statistics (Dmean, Dmin, Dmax, D98%), Paddick conformity index (PCI), Paddick gradient index (GI), ICRU Report 83 homogeneity index (HI), brain V12Gy, and brainstem Dmax. Because plans were generated for the same patients, paired comparisons were performed using two-sided Wilcoxon signed-rank tests (*p* < 0.05). Results: Both HA techniques achieved a higher near-minimum target dose than CK, with significantly higher PTV D98% (CK 12.35 ± 0.52 Gy; HA-SMLC 12.54 ± 0.28 Gy; HA-HDMLC 12.57 ± 0.35 Gy; *p* < 0.05). HA reduced target hotspots, with lower PTV Dmax than CK (CK 15.25 ± 0.32 Gy; HA-SMLC 14.70 ± 0.39 Gy; HA-HDMLC 14.73 ± 0.32 Gy; *p* < 0.05), and improved homogeneity and dose falloff as reflected by HI and GI (*p* < 0.05). CK achieved the highest conformity by PCI (*p* < 0.05), while HA-HDMLC improved PCI compared with HA-SMLC (*p* < 0.05). Brain V12Gy and brainstem Dmax were low and did not differ significantly among techniques. Conclusions: HA provides dosimetric performance comparable to CK for single-fraction VS SRS, with improved near-minimum PTV dose, reduced hotspots, and steeper dose gradients. Although CK showed the highest PCI overall, conformity improved with HA when a high-definition MLC was used. Overall, these findings support HA, particularly HA-HDMLC, as an efficient and clinically practical option for VS SRS treatment planning.

## Simple Summary

Vestibular schwannoma is a benign tumor located near critical structures such as the brainstem and cranial nerves, which makes stereotactic radiosurgery (SRS) planning technically demanding. CyberKnife (CK) is a commonly used radiosurgery platform for vestibular schwannoma, while HyperArc (HA) is a newer automated workflow that delivers noncoplanar volumetric-modulated arc therapy on a standard linear accelerator. In this retrospective dosimetric study, we replanned fifteen CK-treated cases using HA with either a standard or a high definition multileaf collimator and compared plan quality metrics. HA improved target near-minimum dose, reduced hotspots, and achieved a steeper dose falloff, whereas CK provided slightly higher conformity. These findings support automated HA noncoplanar VMAT as an efficient alternative for treating small vestibular schwannomas with single-fraction SRS.

## 1. Introduction

Vestibular schwannoma (VS), also termed acoustic neuroma, is a typically benign, slow-growing tumor that arises from Schwann cells of the vestibular portion of the eighth cranial nerve. VS represents a substantial proportion of cerebellopontine angle tumors and most commonly presents with unilateral sensorineural hearing loss, tinnitus, or imbalance, with symptoms driven by tumor size, intracanalicular extension, and proximity to the brainstem and cranial nerves [1,2].

Management strategies for VS include observation (“wait-and-scan”), microsurgical resection, and radiotherapy delivered as stereotactic radiosurgery (SRS) or stereotactic radiotherapy (SRT) [2,3,4,5]. Treatment selection is individualized and commonly considers patient age and comorbidities, tumor size (often summarized by Koos stage), documented growth on serial imaging, baseline hearing status (e.g., Gardner-Robertson class), cranial nerve symptoms, and patient preference regarding the trade-offs among tumor control, functional preservation, and treatment morbidity [2,3,5].

Single-fraction SRS is widely used for small to moderate VS because it offers durable tumor control with a low incidence of long-term facial or trigeminal neuropathy when contemporary marginal doses are used, commonly in the 12–13 Gy range [3,4,5,6,7]. Modern series report tumor control rates typically in the mid- to high-90% range for small-volume or intracanalicular tumors, while functional hearing preservation varies by baseline hearing, tumor characteristics, and follow-up duration. A recent review summarizes 5-year hearing preservation rates commonly around 57–74%, with further decline at longer follow-up (e.g., 10-year hearing preservation around 24–44% in selected series), emphasizing both the importance and the challenge of long-term functional preservation in a benign population [5]. From a planning perspective, VS SRS is technically demanding because clinically relevant dose metrics are governed by millimeter-scale geometry. Targets are small, irregular, and may extend into the internal auditory canal, while the brainstem, cochlea, and cranial nerves are immediately adjacent. As a result, subtle differences in dose shaping and falloff can alter maximum dose to critical structures or the extent of intermediate-dose spillage into normal brain, even when prescription coverage is matched [3,4].

With recent developments in radiotherapy, multiple radiosurgical delivery platforms are used for intracranial SRS. CyberKnife (CK, Accuray Inc., Sunnyvale, CA, USA) is a robotic linear accelerator (linac) system that delivers many non-isocentric, non-coplanar beams with image guidance, enabling highly conformal dose sculpting around complex targets [8]. CK outcome literature in VS demonstrates high rates of growth control with functional preservation. In a meta-analysis of 11 CK series including 800 patients, the pooled tumor control rate was 96.3%, and hearing preservation was 79.1% among patients with measurable hearing, with low rates of cranial nerve toxicity and cerebellar or brainstem toxicity [9].

HyperArc (HA) (Varian Medical Systems, Palo Alto, CA, USA) is an automated planning and delivery solution for linac-based cranial SRS that uses preconfigured noncoplanar partial arcs, standardized couch and gantry geometries, and template-driven optimization to streamline high quality plan generation and delivery [10,11,12]. The workflow automatically places the isocenter within the patient protection zone, selects collimator angles based on target geometry, and provides a virtual dry-run collision check before optimization to improve delivery safety and efficiency [10,11,12]. In brain metastasis series, HA has produced highly conformal plans with steep gradients while improving planning consistency and treatment efficiency compared with conventional volumetric-modulated arc therapy (VMAT) approaches [13,14]. There is growing clinical interest in whether automated linac-based SRS approaches can provide dosimetric performance comparable to dedicated robotic radiosurgery systems while offering a more standardized and efficient workflow. Prior comparisons between robotic radiosurgery and linac-based noncoplanar VMAT techniques have largely focused on mixed intracranial lesion cohorts, including brain metastases and other benign tumors, where lesion size, number, and spatial distribution can strongly influence normal brain dose and plan quality metrics. Although these studies provide useful context, VS represents a distinct planning challenge because the target is typically extra-axial and often closely abuts the brainstem and cochlea, such that small differences in conformity, hotspot distribution, and dose falloff may be particularly relevant.

Recently, HA has also been applied to benign intracranial tumors because of its favorable dose distribution. In a dosimetric study of benign intracranial lesions comparing HA and cone-based CK, Ho et al. reported that HA could improve conformity and reduce intermediate- and low-dose spillage compared with alternative approaches under matched planning objectives [15]. In a dosimetric study of optic nerve sheath meningioma patients comparing HA with coplanar VMAT techniques, Xiong et al. reported that HA achieved better target coverage and dose falloff while improving organs at risk (OARs) sparing [16]. However, data directly comparing CK and HA specifically for vestibular schwannoma remain limited. The purpose of this study was to compare dosimetric outcomes for single-fraction VS SRS plans created with CK versus automated HA noncoplanar VMAT delivered on a conventional linac, and to evaluate the effect of MLC leaf width by comparing HA plans generated with standard versus high definition MLC configurations.

## 2. Materials and Methods

### 2.1. Patient Selection and Study Design

This retrospective dosimetric study included 15 patients with VS who previously underwent single-fraction SRS using a CK system at our institution between 2021 and 2025. The study was approved by the Institutional Review Board (IRB). All clinical CK plans prescribed 12.5 Gy in one fraction to the planning target volume (PTV). Cases were selected to reflect lesions typically managed with single-fraction SRS in contemporary practice, including small to moderate tumors with a maximum diameter of 2 cm and without significant mass effect [3,4]. Patient demographics and clinical characteristics are summarized in Table 1.

### 2.2. Target and OAR Delineation

Computed tomography (CT) simulations (Brilliance Big Bore, Philips Medical Systems, Nederland B.V., Best, The Netherlands) were acquired with 1 mm slice thickness and a 512 × 512 matrix. Targets and OARs were delineated on the original simulation datasets used for clinical treatment planning. Magnetic resonance imaging (MRI) was registered to the CT to aid target definition. The gross tumor volume (GTV) was defined as the contrast-enhancing schwannoma, and the PTV was set equal to the GTV. The median PTV was 0.38 cm^3^ (range: 0.15 to 2.22 cm^3^). Target volumes and key OARs, including the brainstem and brain, were contoured by an experienced radiation oncologist at our institution.

### 2.3. CK Planning

Clinical CK plans were created using the institution’s CK planning system with inverse planning for a large number of noncoplanar, non-isocentric beam directions delivered by a robotic CK linac. The treatment plans for the 15 patients treated with CK (M6, Accuray Inc., Sunnyvale, CA, USA) were analyzed without modification. All patients were simulated head-first in the supine position and immobilized with a pre-molded U-Frame mask. Plans were generated in Accuray Precision (v2.0.1.1) using 6 MV flattening filter free (FFF) photon beams with fixed cones, delivered in a non-isocentric arrangement with beam nodes and cone sizes selected to optimize target coverage and OAR sparing (for example, the brainstem). A total of 39 to 58 beam nodes were used (median 49). Planning optimization used the VOLO algorithm, and dose calculation used the ray-tracing algorithm in high-resolution mode with a dose grid of 0.1 × 0.1 × 0.1 cm^3^. Because the CK plans were clinically delivered, they were treated as the reference standard for comparison.

### 2.4. HA Planning and MLC Configurations

**Figure 1:**
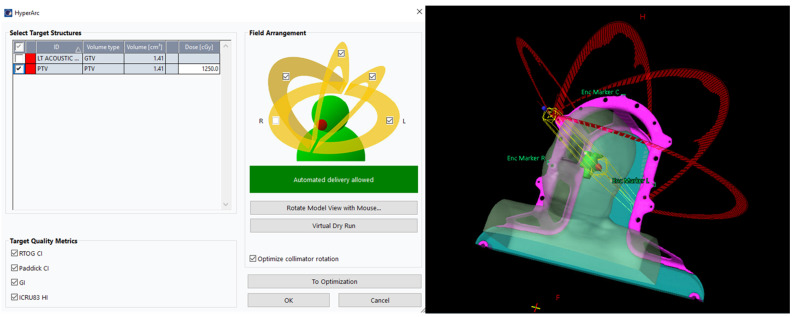
Screenshot of the HA planning interface in Eclipse. The virtual dry-run feature allows collision checking for selected arcs prior to optimization.

Each case was replanned using the Eclipse treatment planning system (Eclipse 16.1; Varian Medical Systems, Palo Alto, CA, USA) with HA. A virtual Encompass (QFix, Avondale, PA, USA) mask was included for all HA plans. HA plans used four preconfigured noncoplanar partial arcs delivered on a conventional linac, with standardized couch and gantry trajectories selected by the HA workflow. Specifically, four half-arcs were used with couch rotations of 0°, 45°, 90° for left-sided VS or 270° for right-sided VS, and 315°. Isocenter placement and collimator angle selection were automatically generated based on target geometry (Figure 1). All HA plans used 6 MV flattening filter free (FFF) photon beams at 1400 monitor units (MU)/min on a Varian TrueBeam linac (Varian Medical Systems, Palo Alto, CA, USA). Two MLC configurations were evaluated: (i) a standard MLC with 40 leaf pairs of 5 mm leaf width in the central region and 20 leaf pairs of 10 mm leaf width in the peripheral region (HA-SMLC) and (ii) a high definition MLC with 32 leaf pairs of 2.5 mm leaf width in the central region and 28 leaf pairs of 5 mm leaf width in the peripheral region (HA-HDMLC). Dose calculation in Eclipse used the Anisotropic Analytical Algorithm (AAA, v16.1) with a 1.25 mm calculation grid. The same planning template and optimization strategy were applied across cases; minor objective adjustments were allowed when needed to satisfy OAR constraints while maintaining target coverage. For a fair dosimetric comparison, the HA plans were normalized to match the prescription isodose target coverage of the corresponding CK plan for each patient. Specifically, normalization was based on PTV V_100%_, defined as the percentage of the PTV receiving 100% of the prescription dose (12.5 Gy).

### 2.5. Dosimetric Parameters and Plan Quality Analysis

Plan evaluation was performed using dosimetric parameters derived from dose-volume histograms (DVHs) for the PTV and OARs. Target endpoints included prescription coverage, near-minimum dose (PTV D_98%_), minimum dose (PTV D_min_), maximum dose (PTV D_max_), and mean dose (PTV D_mean_). Hotspot behavior was characterized using PTV D_max_. Normal tissue endpoints included brain V_12Gy_ (volume receiving at least 12 Gy) and brainstem D_max_. Although V_12Gy_ is most commonly discussed in the setting of intra-axial brain metastasis SRS, it remains a useful summary metric for intermediate-dose spillage in cranial stereotactic planning [17,18].

The Paddick Conformity Index (PCI) [19] was calculated for each plan as follows:

(1) $$PCI=\frac{\left(TV\cap PIV{)}^{2}\right.}{TV\times PIV}$$

where TV is the target volume (PTV), PIV is the volume that receives the prescription dose, and TV ∩ PIV is the portion of the target covered by the prescription isodose. PCI characterizes how precisely the prescription dose conforms to the target, with an ideal value of 1.

Dose homogeneity was quantified using the International Commission on Radiation Units and Measurements (ICRU) Report 83 homogeneity index (HI) [20]:

(2) $$HI=\frac{D_{2\%}-D_{98\%}}{D_{50\%}}$$

where D_2%_ is the dose received by 2% of the PTV, D_98%_ is the dose received by 98% of the PTV, and D_50%_ is the dose received by 50% of the PTV. Lower HI values indicate a more homogeneous dose distribution, with an ideal value of 0.

Dose falloff was assessed using the Paddick Gradient Index (GI) [21]:

(3) $$GI=\frac{PIV_{50\%}}{PIV_{100\%}}$$

where PIV_50%_ is the volume encompassed by the 50% prescription isodose and PIV_100%_ is the volume encompassed by the prescription isodose. GI characterizes the steepness of the dose gradient outside the target; lower GI values indicate sharper dose falloff.

### 2.6. Statistical Analysis

Because all treatment plans were generated for the same patients, dosimetric comparisons were treated as paired data. Pairwise comparisons between CK, HA-SMLC, and HA-HDMLC were performed using two-sided Wilcoxon signed-rank tests (CK vs. HA-SMLC, CK vs. HA-HDMLC, and HA-SMLC vs. HA-HDMLC). This nonparametric paired test was selected because the sample size was relatively small and the planning data were matched within each patient. A *p*-value less than 0.05 was considered statistically significant. Because this was an exploratory retrospective dosimetric-planning study with a limited sample size and several correlated dosimetric endpoints, no formal multiple-comparison correction was applied. Therefore, pairwise *p*-values, particularly borderline results, should be interpreted cautiously.

## 3. Results

All 15 cases were successfully replanned using the HA workflow with both MLC configurations. Table 2 summarizes the dosimetric comparisons among CK, HA-SMLC, and HA-HDMLC plans for target coverage, plan quality indices, and normal tissue sparing. Significant technique-dependent differences were observed in target dose distribution and dose falloff.

**Figure 2:**
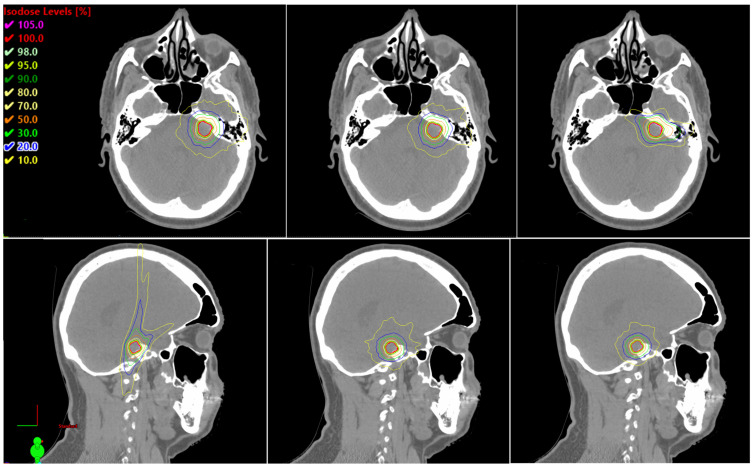
Representative axial (**top row**) and sagittal (**bottom row**) isodose distributions for a VS case planned with CK (**left**), HA-SMLC (**middle**), and HA-HDMLC (**right**). The PTV is outlined in red. Isodose lines are expressed as percentage of the prescription dose to illustrate differences in conformity and intermediate-dose spillage across techniques.

**Figure 3:**
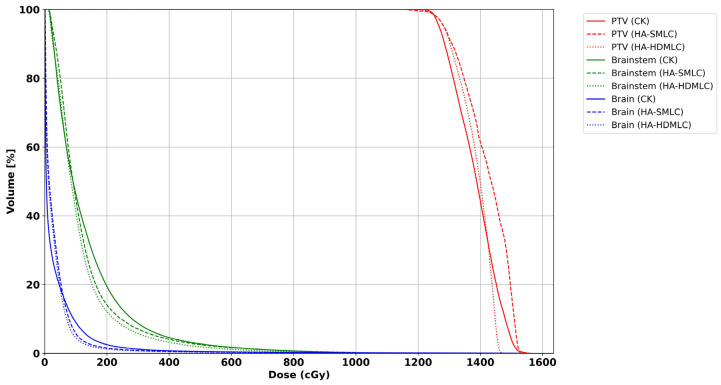
DVHs for the PTV and selected OARs for the patient shown in Figure 2. Solid line = CK, dashed line = HA-SMLC, dotted line = HA-HDMLC.

Representative axial and sagittal isodose distributions for one typical VS case are shown in Figure 2 (CK left, HA-SMLC middle, HA-HDMLC right). In this example, both HA plans show a more uniform high-dose region within the PTV and a more compact intermediate dose envelope, consistent with lower D_max_ and improved HI in Table 2. The 50% isodose volume is smaller with HA, aligning with the lower GI values and indicating steeper dose falloff. Prescription isodose coverage remains comparable across techniques, although CK shows slightly tighter conformity around the target edge, consistent with the higher PCI. Figure 3 presents DVHs for the PTV and selected OARs for the same patient (solid = CK, dashed = HA-SMLC, dotted = HA-HDMLC). The PTV DVHs demonstrate higher D_98%_ and a reduced high-dose tail with HA, while the OAR DVHs show similar brain intermediate dose exposure and modest differences in brainstem dose that mirror the cohort trends in Table 2.

### 3.1. Target Coverage and Dose Statistics

**Figure 4:**
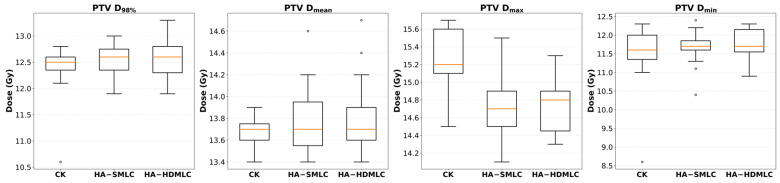
Box plots of PTV D_98%_, D_mean_, D_max_, and D_min_ for CK, HA-SMLC, and HA-HDMLC plans.

Figure 4 summarizes PTV dose statistics. Prescription coverage was matched by design; however, both HA plans achieved higher PTV D_98%_ than CK (CK: 12.35 ± 0.52 Gy; HA-SMLC: 12.54 ± 0.28 Gy, *p* = 0.005; HA-HDMLC: 12.57 ± 0.35 Gy, *p* = 0.004), indicating an improved near-minimum target dose and fewer low-end outliers. PTV D_mean_ was similar among techniques (CK: 13.67 ± 0.13 Gy; HA-SMLC: 13.77 ± 0.34 Gy; HA-HDMLC: 13.82 ± 0.36 Gy; all pairwise *p* > 0.05), suggesting that differences were driven by peripheral coverage and hotspot modulation rather than a systematic shift in average target dose.

Both HA techniques reduced hotspot magnitude, with significantly lower PTV D_max_ than CK (CK: 15.25 ± 0.32 Gy; HA-SMLC: 14.70 ± 0.39 Gy, *p* = 0.002; HA-HDMLC: 14.73 ± 0.32 Gy, *p* = 0.002). This reduction is visible in Figure 4 as a downward shift in the D_max_ distributions for the HA plans. PTV D_min_ was numerically higher for HA, but the difference was not statistically significant (CK: 11.49 ± 0.89 Gy; HA-SMLC: 11.63 ± 0.46 Gy, *p* = 0.800; HA-HDMLC: 11.73 ± 0.42 Gy, *p* = 0.201).

### 3.2. Conformity, Homogeneity, and Dose Gradient

**Figure 5:**
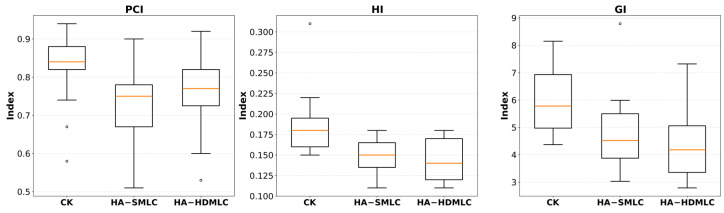
Box plots of conformity (PCI), homogeneity (HI), and gradient (GI) indices for CK, HA-SMLC, and HA- HDMLC plans.

Plan quality indices are visualized in Figure 5. Dose homogeneity within the PTV improved with HA, with both HA techniques demonstrating significantly lower HI than CK (CK: 0.19 ± 0.04; HA-SMLC: 0.15 ± 0.02, *p* = 0.001; HA-HDMLC: 0.14 ± 0.02, *p* = 0.001). This finding is consistent with the combined effect of higher D_98%_ and lower D_max_ in the HA plans, and the HI boxplots show a lower median and reduced spread for both HA techniques relative to CK.

Dose falloff outside the target was also improved with HA, as demonstrated by significantly lower GI values for HA-SMLC and HA-HDMLC compared with CK (CK: 5.97 ± 1.24 vs. HA-SMLC: 4.79 ± 1.45, *p* < 0.001; vs. HA-HDMLC: 4.37 ± 1.23, *p* < 0.001). Relative to CK, HA-HDMLC reduced GI by 26.8%, indicating a steeper gradient and reduced intermediate-dose spillage; HA-HDMLC also achieved a significantly lower GI than HA-SMLC (*p* < 0.001), suggesting an additional gradient advantage with the high-definition MLC configuration.

Conformity, as measured by the PCI, favored CK. CK achieved the highest PCI (CK: 0.83 ± 0.10), while both HA configurations were lower (HA-SMLC: 0.74 ± 0.11, *p* = 0.005 vs. CK; HA-HDMLC: 0.77 ± 0.11, *p* = 0.026 vs. CK). CK showed the highest PCI overall, whereas HA-HDMLC improved PCI relative to HA-SMLC. Given the number of pairwise comparisons performed, the difference between CK and HA-HDMLC should be interpreted with caution.

### 3.3. OAR Dose Metrics

**Figure 6:**
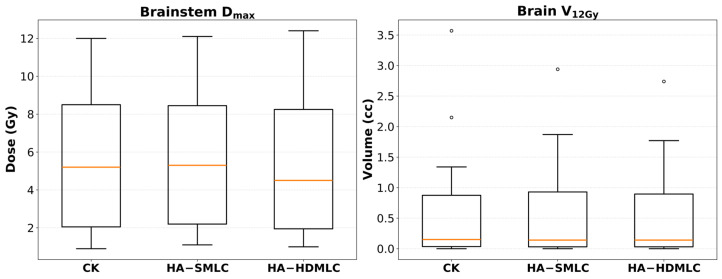
Box plots of brainstem D_max_ and normal brain V_12Gy_ for CK, HA-SMLC, and HA-HDMLC plans.

Normal tissue endpoints are shown in Figure 6. Brain V_12Gy_ was low across all three techniques and did not differ significantly in pairwise testing (CK: 0.68 ± 1.01 cm^3^ vs. HA-SMLC: 0.63 ± 0.86 cm^3^, *p* = 0.767; vs. HA-HDMLC: 0.60 ± 0.81 cm^3^, *p* = 0.475). The V_12Gy_ distributions in Figure 6 demonstrate that most cases clustered near small volumes, with occasional higher outliers that reflect patient-specific anatomy rather than a consistent technique-driven effect; there was a trend toward lower V_12Gy_ with HA-HDMLC compared with HA-SMLC, but it did not reach statistical significance (*p* = 0.063).

Brainstem D_max_ exhibited substantial interpatient variability across all techniques (CK: 5.48 ± 3.69 Gy; HA-SMLC: 5.55 ± 3.76 Gy; HA-HDMLC: 5.15 ± 3.78 Gy). Differences between CK and either HA technique were not statistically significant (CK vs. HA-SMLC *p* = 0.454; CK vs. HA-HDMLC *p* = 0.158). However, HA-HDMLC yielded a significantly lower brainstem D_max_ than HA-SMLC (*p* = 0.006), suggesting a modest brainstem-sparing advantage with the high definition MLC configuration in this cohort. Importantly, all plans met institutional brainstem constraints for single-fraction VS SRS.

## 4. Discussion

SRS is an established primary treatment modality for small- to moderate-sized VS [5]. Planning must balance durable tumor control with preservation of adjacent neural structures, and because VS targets commonly abut the brainstem, modest differences in conformity, dose falloff, and hotspot location can be clinically meaningful even when prescription coverage is matched [3,4,5]. Most platform comparisons of automated noncoplanar VMAT and robotic SRS focus on brain metastases or mixed intracranial cohorts, in which lesion number, size, and spatial distribution strongly influence normal-brain dose and intermediate-dose spillage [11,12,22,23]. Even so, prior work consistently shows that stereotactic plan quality depends on lesion size, optimizer configuration, and delivery geometry, and that automated noncoplanar arc delivery can improve workflow efficiency while maintaining high-quality stereotactic dosimetry [11,12,22,23]. Within benign disease, Ho et al. compared HA and cone-based CK under matched stereotactic objectives and reported favorable intermediate-and low-dose spillage metrics for HA while maintaining high target coverage [15], and Hotsinpiller et al. demonstrated clinically feasible frameless HA delivery for benign intracranial tumors with short treatment times and low rates of severe toxicity [24]. To our knowledge, the present study is the first vestibular schwannoma-specific dosimetric comparison of three representative single-fraction SRS planning techniques, including CK, HA-SMLC, and HA-HDMLC. Across 15 single-fraction cases prescribed 12.5 Gy, all plans were clinically acceptable. HA achieved dosimetric performance comparable to clinically delivered CK plans, with consistent improvements in near-minimum target dose, hotspot reduction, homogeneity, and dose gradient, while CK maintained higher conformity by PCI.

The higher PTV D_98%_ observed with HA indicates more robust near-minimum dose to the target at a matched prescription coverage level. In VS, irregular intracanalicular extension and concave target and OAR interfaces can increase the risk of peripheral underdosage when optimization is strongly constrained by brainstem proximity. In our cohort, both HA techniques increased D_98%_ relative to CK, suggesting a more consistent low dose tail of the PTV DVH. HA also reduced hotspot magnitude, with lower PTV D_max_ compared with CK. While some intratarget heterogeneity is expected and often acceptable in SRS, limiting excessive hotspots may be desirable when high-dose regions occur near critical neural structures. Current practice emphasizes conservative marginal-dose selection, commonly 12 to 13 Gy, to preserve cranial nerve function [3,5], and within that framework, reducing extreme hotspots may provide an additional geometry-dependent safety margin when hotspots spatially coincide with vulnerable structures [4,6]. However, although several dosimetric differences between CK and HA reached statistical significance, their clinical significance remains uncertain. The absolute increase in PTV D_98%_ with HA was modest and should not be interpreted as evidence of a meaningful tumor control advantage, and the clinical impact of reduced hotspot magnitude cannot be determined from this dosimetric study alone. Therefore, these findings are best interpreted as demonstrating planning and dosimetric differences between techniques rather than clinical superiority of one platform over another.

CK’s higher PCI in this cohort likely reflects the extensive noncoplanar sampling achievable with robotic, non-isocentric delivery [8]. In contrast, HA relies on a limited set of standardized noncoplanar arcs that are designed to balance collision safety with delivery efficiency. While this standardization can streamline planning, it may also limit conformity for very small, elongated, or highly eccentric targets. Despite this, HA produced a steeper dose falloff outside the target, as demonstrated by significantly lower GI values compared with CK. This finding should be interpreted cautiously, as it may reflect the matched-coverage normalization strategy used in this cohort as well as differences in beam shaping and optimization between cone-based CK and MLC-based HA plans. Thus, the lower GI observed with HA should not be viewed as a universal platform advantage, but rather as a result influenced by planning approach and hardware characteristics. In the setting of VS, where the brainstem is frequently the dominant limiting organ, improved gradient may be clinically relevant because it can reduce intermediate-dose spillage toward the brainstem while maintaining robust target coverage. Thus, although conformity remains important, rapid dose falloff may be particularly critical in VS when the tumor closely abuts the brainstem or cochlea. In addition to gradient, HA improved dose homogeneity within the target. Both HA configurations demonstrated significantly lower HI than CK, indicating a narrower separation between near-maximum and near-minimum PTV dose. This finding is consistent with the concurrent increase in D_98%_ and reduction in D_max_ observed with HA, and it suggests that the automated noncoplanar arc approach produced a more uniform internal dose distribution while still maintaining stereotactic dose falloff. Although greater intratarget heterogeneity is sometimes considered desirable in SRS, this may be more relevant for malignant lesions than for benign vestibular schwannoma. In VS, where treatment aims to preserve hearing and cranial nerve function, lower HI with reduced hotspot magnitude may be favorable when high-dose regions lie near critical neural structures. Therefore, the improved homogeneity observed with HA should not necessarily be viewed as a disadvantage, although its clinical significance cannot be determined from this dosimetric study alone.

MLC leaf width influenced conformity in the expected direction. HA-HDMLC achieved a significantly higher PCI than HA-SMLC, consistent with improved geometric approximation of compact and highly curved target boundaries [10,12]. In our cohort, this increase in PCI with the high-definition MLC occurred alongside improved gradient performance, with HA-HDMLC also demonstrating a lower GI than HA-SMLC, suggesting that finer leaf resolution can improve both conformity and dose falloff within the HA framework. These observations align with prior benign-lesion replanning studies comparing HA and CK, which emphasize that delivery geometry and optimizer configuration can meaningfully affect conformity and intermediate-dose spillage. This reinforces the importance of technique-specific tuning and MLC selection when optimizing stereotactic plan quality [15].

Brain V_12Gy_ was low across all techniques and did not differ significantly. In our cohort, mean V_12Gy_ remained under 1 cm^3^ for all three approaches, and the boxplots suggested that most cases clustered near very small volumes, with a small number of higher outliers. The higher Brain V_12Gy_ outliers observed in the CK group were likely related to case-specific anatomy and target geometry, rather than a systematic platform-dependent effect. This pattern indicates that intermediate-dose exposure to the brain was generally limited for VS SRS in our cohort, and it also suggests that patient-specific anatomy and target size, rather than treatment technique alone, likely drove the higher brain V_12Gy_ cases. Although HA improved GI, the lack of separation in V_12Gy_ is plausible because V_12Gy_ is a relatively high-dose threshold near the prescription dose for a 12.5 Gy plan, and it is therefore more sensitive to the size and shape of the prescription isodose volume than to differences in lower-dose spread captured by gradient metrics [15,17,18].

Brainstem D_max_ remains a key OAR endpoint in VS because targets often abut the brainstem in the cerebellopontine angle. In our study, brainstem D_max_ showed substantial interpatient variability and did not differ significantly between CK and either HA technique. However, HA-HDMLC demonstrated a lower brainstem D_max_ than HA-SMLC on pairwise testing, suggesting that finer leaf resolution may provide a modest advantage for select anatomies when the brainstem lies close to the high-dose gradient.

Workflow considerations can influence technology adoption and were a key motivation for this comparison. CK typically delivers many beams sequentially, whereas HA delivers a small number of noncoplanar VMAT arcs, which can shorten beam on time and overall treatment time and reduce the window for intrafraction motion. Prior intracranial SRS comparisons have reported shorter treatment times with HA, a potential advantage for single-fraction workflows where motion can have a disproportionate dosimetric impact [11,12,22,23]. In a frameless HA series for benign intracranial tumors, Hotsinpiller et al. reported a mean beam-on time of about 2 min and a mean overall treatment time of about 10 min, supporting efficient linac-based delivery in benign disease [24]. Consistent with this, in our cohort, HA plans were also associated with substantially lower monitor units and shorter treatment times than CK. Although delivery efficiency was not a primary endpoint of this study, these findings provide additional practical support for the workflow advantages of HA.

Motion management remains relevant for frameless cranial SRS, particularly for single-fraction treatments. Surface-guided radiotherapy (SGRT) systems such as VisionRT are increasingly used in cranial stereotactic workflows to provide continuous monitoring and facilitate rapid detection of intrafraction motion [25,26,27]. HA can be delivered with the Encompass (QFix, Avondale, PA, USA) frameless mask system and paired with SGRT for immobilization, setup guidance, and intrafraction tracking [28,29,30]. Prior studies have reported submillimeter translational accuracy, subdegree rotational stability, and preserved treatment efficiency with these approaches during frameless intracranial SRS [25,31,32,33]. Although such workflow features may support efficient clinical implementation and reduce the need for repeated cone-beam CT (CBCT) verification in some cases, they were not directly evaluated in the present study and therefore remain outside the primary scope of this dosimetric comparison.

This study has several limitations. First, although 15 patients are acceptable for a treatment planning comparison study, it remains a relatively small cohort and may limit statistical power, particularly for detecting subtle differences between techniques. Second, multiple pairwise comparisons were performed across several correlated dosimetric endpoints without formal multiplicity adjustment, so an increased risk of type I error cannot be excluded, particularly for borderline significant findings. However, the main conclusions are supported by consistent trends across multiple related endpoints rather than isolated marginal results. Larger studies are needed to confirm the robustness of these findings. Third, these results may not extend to larger VS, postoperative targets, or cases treated with hypofractionated regimens. Hypofractionated stereotactic radiotherapy, such as 25 Gy in five fractions, is commonly used for larger tumors or lesions that closely abut the brainstem to improve tolerance, and contemporary series support this approach with high tumor control and low rates of serious cranial nerve toxicity [5,34]. Future work should therefore expand the comparison to hypofractionated cohorts and incorporate regimen specific planning priorities and constraints. Fourth, our dosimetric evaluation focused on a limited set of OAR endpoints, namely brainstem D_max_ and brain V_12Gy_. Other clinically relevant VS-related structures, including the cochlea, trigeminal nerve, and facial nerve, were not included in the formal analysis because their doses were generally low and below clinical constraints in this cohort. Nevertheless, broader OAR reporting would strengthen the clinical applicability of future VS-focused dosimetric studies. An additional limitation is that CK and HA plans were calculated using different dose algorithms and grid resolutions. CK used Ray-Tracing with a 1.0 mm grid, whereas HA used AAA with a 1.25 mm grid. For small posterior fossa targets near bone and air interfaces, these differences may affect dosimetric parameters such as D_min_, D_98%_, and D_max_. Therefore, some of the observed differences may reflect calculation method as well as platform-specific planning and delivery characteristics. Finally, because the CK plans were clinically delivered while the HA plans were generated retrospectively, planner experience, institutional templates, and technique specific planning habits may have influenced the results. Standardized planning protocols across modalities would help reduce this source of variability. Future comparisons may also benefit from composite endpoints that capture both plan quality and deliverability by combining PCI and GI and key OAR metrics with delivery time measurements and motion data to better characterize the overall clinical risk profile of each modality.

## 5. Conclusions

For small VS treated with single-fraction SRS, automated HA noncoplanar VMAT achieved dosimetric performance comparable to CK, with higher near-minimum target dose, fewer hotspots, improved homogeneity, and steeper dose gradients. CK showed the highest PCI numerically, while conformity with HA improved when a high-definition MLC was used. HA also offers a standardized, template-based workflow with streamlined planning and automated noncoplanar delivery. When paired with frameless immobilization and optional surface guided imaging, it supports accurate and efficient cranial SRS delivery. Overall, these findings support HA, particularly HA-HDMLC, as a practical treatment planning approach for VS SRS; however, the clinical significance of the observed dosimetric differences remains uncertain and should be validated in future outcome-based studies. 

## Figures and Tables

**Table 1:** Summary of clinical characteristics for 15 patients with VS.

Characteristics	
Patient number	15
Gender	
Male	5
Female	10
Age (y)	
Median	64
Range	39–77
Side	
Left	7
Right	8
PTV (cm^3^)	
Median	0.38
Range	0.15–2.22

**Table 2:** Dosimetric comparison among CK, HA-SMLC, and HA-HDMLC plans for PTV coverage, plan quality indices, and OAR endpoints. Values are presented as mean ± standard deviation (SD). * Statistically significant with *p* value threshold of 0.05.

Structure	Dosimetric Parameters	Technique						*p*-Value	*p*-Value	*p*-Value
		CK (a)		HA-SMLC (b)		HA-HDMLC (c)		a vs. b	a vs. c	b vs. c
		Mean	Std	Mean	Std	Mean	Std			
PTV	D_98%_ (Gy)	12.35	0.52	12.54	0.28	12.57	0.35	0.005 *	0.004 *	0.198
	D_mean_ (Gy)	13.67	0.13	13.77	0.34	13.82	0.36	0.181	0.090	0.437
	D_max_ (Gy)	15.25	0.32	14.70	0.39	14.73	0.32	0.002 *	0.002 *	0.724
	D_min_ (Gy)	11.49	0.89	11.63	0.46	11.73	0.42	0.800	0.201	0.238
	Paddick CI	0.83	0.10	0.74	0.11	0.77	0.11	0.005 *	0.026 *	0.001 *
	ICRU83 HI	0.19	0.04	0.15	0.02	0.14	0.02	0.001 *	0.001 *	0.504
	GI	5.97	1.24	4.79	1.45	4.37	1.23	0.000 *	0.000 *	0.000 *
Brainstem	D_max_ (Gy)	5.48	3.69	5.55	3.76	5.15	3.78	0.454	0.158	0.006 *
Brain	V_12Gy_ (cm^3^)	0.68	1.01	0.63	0.86	0.60	0.81	0.767	0.475	0.063

## Data Availability

The data used and analyzed during the current study are available from the corresponding authors upon reasonable request.

## Abbreviations

The following abbreviations are used in this manuscript:

AAA	Anisotropic Analytical Algorithm
ANOVA	Analysis of variance
CBCT	Cone-beam computed tomography
CI	Conformity index
CK	CyberKnife
CT	Computed tomography
DVH	Dose-volume histogram
FFF	Flattening filter free
GI	Gradient index (Paddick gradient index)
GTV	Gross tumor volume
HA	HyperArc
HA-HDMLC	HyperArc using a 2.5 mm high-definition MLC
HA-SMLC	HyperArc using a 5.0 mm standard MLC
HI	Homogeneity index
ICRU	International Commission on Radiation Units and Measurements
IRB	Institutional Review Board
MLC	Multileaf collimator
MRI	Magnetic resonance imaging
MU	Monitor units
MV	Megavoltage
OAR	Organ at risk
PCI	Paddick conformity index
PTV	Planning target volume
SGRT	Surface-guided radiotherapy
SRS	Stereotactic radiosurgery
SRT	Stereotactic radiotherapy
VMAT	Volumetric-modulated arc therapy
VS	Vestibular schwannoma
